# Prognostic factors in the treatment of polypoidal choroidal vasculopathy with conbercept: a post hoc analysis of the STAR study

**DOI:** 10.1186/s40662-025-00441-5

**Published:** 2025-06-18

**Authors:** Tsung-I. Wang, Jinfeng Qu, Ran Tang, Xuan Shi, Xin Ying, Ye Tao, Xiaoxin Li

**Affiliations:** 1 Department of Ophthalmology, Peking University People’s Hospital, Beijing, China; 2 Beijing Key Laboratory of Ocular Disease and Optometry Science, Peking University People’s Hospital, Beijing, China; 3 Xiamen Eye Center, Xiamen University, Xiamen, China

**Keywords:** Anti-vascular endothelial growth factor, Conbercept, Polypoidal choroidal vasculopathy, Neovascular age-related macular degeneration, Treat-and-extend

## Abstract

**Background:**

A post hoc analysis of the STAR study, which was a 48-week, phase IV, multicenter randomized controlled multicenter clinical trial was performed. This study aims to identify the baseline factors associated with visual and anatomic changes over 48 weeks in the treatment of active polypoidal choroidal vasculopathy (PCV) with conbercept.

**Methods:**

In the STAR study, 249 participants were randomized to either the 3 + Q12W (3 monthly injections followed by injections every 12 weeks) or 3 + TAE (3 monthly injections followed by treat and extend regimen) group. The association of 27 baseline factors with three outcomes—changes in best-corrected visual acuity (BCVA), central retinal thickness (CRT), and maximum retinal thickness (MRT) from baseline to 48 weeks—was investigated using univariate regression analysis followed by multivariate linear regression analysis.

**Results:**

The final multivariate model indicated that worse baseline BCVA (*P* < 0.01), CRT ≤ 400 μm (*P* < 0.01), fewer polypoidal lesions (*P* < 0.01), and younger age at baseline (*P* = 0.04) were associated with greater BCVA gain at week 48. Higher CRT and MRT at baseline were associated with a greater reduction in CRT and MRT at week 48, separately (*P* < 0.01 and *P* < 0.01, respectively). Smaller pigment epithelial detachment (PED) volume at baseline was associated with greater reductions in CRT and MRT at week 48 (both *P* < 0.01). Eyes with relatively good BCVA (> 73 letters) at baseline exhibited lower reductions in CRT and MRT at week 48 (*P* < 0.01 and *P* = 0.02, respectively). At week 48, eyes with hemorrhagic PEDs showed greater reductions in CRT and MRT than those with fibrovascular PEDs (*P* = 0.02 and *P* = 0.03, respectively). Furthermore, eyes with shallow irregular or sharp-peaked PEDs exhibited greater reductions in CRT (both *P* < 0.01) and MRT (*P* = 0.01 and *P* < 0.01, respectively) than those with multilobular PEDs from baseline to week 48.

**Conclusions:**

In Chinese patients with PCV receiving intravitreal injections of conbercept, baseline characteristics, including age, BCVA, CRT, MRT, number of polypoidal lesions, PED volume, and PED types and morphology, served as predictors of visual and anatomical changes over 48 weeks.

**Supplementary Information:**

The online version contains supplementary material available at 10.1186/s40662-025-00441-5.

## Background

Polypoidal choroidal vasculopathy (PCV) is recognized as a subtype of macular neovascularization (MNV), characterized by the presence of subretinal orange-red nodular lesions, choroidal branching neovascular networks, and vascular polypoidal lesions [1]. Recent studies have revealed a close association between PCV and dilated hyperpermeable choroidal vessels, regardless of accompanying choroidal thickening, indicating the classification of PCV within the pachychoroid spectrum [2, 3]. PCV is notably more prevalent in individuals aged 55–70 years, particularly among Asian populations [4, 5].

Currently, intravitreal injection (IVI) of anti-vascular endothelial growth factor (anti-VEGF), known as anti-VEGF therapy, has replaced photodynamic therapy (PDT) as the first-line treatment for neovascular age-related macular degeneration (nAMD) and has demonstrated effective results in treating active PCV [6–8] Several clinical trials have shown that anti-VEGF monotherapy, including aflibercept and ranibizumab, yields favorable visual outcomes for PCV compared with PDT or combination therapy [9–12]. Furthermore, compared with PDT, anti-VEGF therapy is associated with a lower risk of massive macular hemorrhage, retinal pigment epithelium (RPE) tear, and other complications [13]. Several factors have been reported to influence the outcomes of anti-VEGF therapy for nAMD, including age, baseline subretinal fluid (SRF) levels or pigment epithelial detachment (PED) volume, baseline best-corrected visual acuity (BCVA) or central retinal thickness (CRT), and size of choroidal neovascularization (CNV). However, it remains unclear whether these factors are similarly correlated with the outcomes of anti-VEGF therapy for PCV [14–17].

Conbercept (Lumitin®, Chengdu Kanghong Biotech Co., Ltd., P.R. China), a novel fusion protein that inhibits the activity of VEGF-A, VEGF-B, and placenta growth factor, was approved for the treatment of MNV in China in 2013 [18–20]. Most reports regarding its efficacy for PCV are based on retrospective studies, which show variability in outcomes amongst them [21–23]. Moreover, the prognostic factors associated with IVI of conbercept for PCV have not yet been reported.

The STAR study is the largest multicenter randomized controlled trial (RCT) comparing the treat-and-extend (TAE) regimen with a fixed dosing regimen of IVI of conbercept in patients with treatment-naïve PCV in China. At the 48-week visit, both regimens demonstrated substantial improvements in BCVA, CRT, maximum retinal thickness (MRT), PED height and volume, branching vascular network (BVN) area, number and area of polypoidal lesions, and rate of complete polypoidal lesion regression, with no significant differences between the two approaches [24]. Therefore, understanding the baseline factors associated with the outcomes of conbercept treatment (IVI) for PCV is crucial for refining expectations for both patients and physicians.

This post hoc analysis of data from the STAR study explores the potential baseline factors that impact the visual and anatomic prognosis of conbercept treatment (IVI) for PCV at week 48.

## Methods

### Overview of the STAR study

In the STAR study, 249 patients from 39 sites across China were enrolled. The study adhered to the principles of the Declaration of Helsinki and was approved by the by the Ethnic Committee of People’s Hospital of Peking University (2014PHA044-02). Written informed consent was obtained from all participants. The methods and detailed protocol of the STAR study have been published online [24]. Briefly, participants aged more than 45 years with treatment-naïve active PCV involving the fovea, with CRT ≥ 250 μm and MRT < 600 μm (if sub-RPE or subretinal hemorrhage was present), were included. BCVA ranged from 19 to 83 ETDRS letters (Snellen equivalent: 20/400 to 20/25). The enrolled patients (one eye per patient) were randomized in a 1:1 ratio to either the 3 + Q12W or 3 + TAE group. Following 3 monthly injections of 0.5 mg intravitreal conbercept, the 3 + Q12W group received regular injections every 12 weeks, whereas the 3 + TAE group received a TAE regimen. During follow-up in the 3 + Q12W group, additional injections were administered as needed according to the protocol. PDT was administered if the protocol criteria were met at the 24-week visit in both groups.

### Image grading

Optical coherence tomography (OCT), color fundus photography (CFP), indocyanine green angiography (ICGA), and fluorescein fundus angiography images from all participants were collected and independently assessed by two graders. In cases of disagreement, the graders convened for an open adjudication to reach a consensus. If they could not agree, a third senior ophthalmologist made the final decision.

The area of polypoidal lesions, area of BVNs, and the greatest linear dimension (GLD) of the entire lesion were measured on ICGA using the software’s built-in caliper (Spectralis, Heidelberg Engineering, Heidelberg, Germany). The area of retinal hemorrhage was measured on CFP using the built-in measuring tools of CFP instruments (Topcon TRC 50-DX, Topcon TRC-50EX, or Zeiss FF 450 plus). CRT was defined as the mean distance between the internal limiting membrane (ILM) and Bruch’s membrane within the 1-mm central subfield of the ETDRS grid. MRT was defined as the maximum distance between the ILM and Bruch’s membrane within the scanned area on OCT. Subfoveal choroidal thickness (SFCT) was measured as the distance between Bruch’s membrane and the chorioscleral interface at the foveal center, using the built-in caliper of the OCT instrument. CRT, MRT, PED volume, and maximum PED height were measured using grading software 3D-OCTOR developed by the Doheny Image Reading Center which was described and validated in our previous publication [24]. Choroidal vascular hyperpermeability (CVH) was defined as multifocal areas of hyperfluorescence with blurred margins during the mid and late phases on ICGA [25–27]. PED types were classified as fibrovascular, hemorrhagic, or serous based on the internal reflection between the RPE and Bruch’s membrane on spectral-domain OCT. PED morphology was categorized as dome-shaped, multilobular, sharp-peaked, shallow irregular, or suspect for RPE tear. When multiple types and shapes of PED are present in a single eye of participants, only the PED closest to the central subfield was evaluated.

### Statistical analysis

The eyes of all patients who completed the 48-week visit were included in the post hoc analysis. Associations between 27 baseline factors and three outcomes—changes in BCVA, CRT, and MRT from baseline to week 48—were investigated. The factors examined were sex, age, treatment regimens, BCVA, CRT, MRT, SFCT, presence of retinal hemorrhage, large hemorrhage (more than 1 disc diameter), intraretinal fluid (IRF), SRF, BVNs, presence of CVH in the scanned area, presence of PED, polypoidal lesions within the 1-mm central subfield, PED types, PED morphology, maximum PED height, PED volume, number of polypoidal lesions, area of polypoidal lesions, BVN area, retinal hemorrhage area, and the GLD of the entire lesion. Univariate regression analysis was initially conducted to identify potential predictors of each outcome. Multicollinearity was assessed by calculating variance inflation factor (VIF) values for all independent variables. Factors with *P* < 0.2 in the univariate analysis, along with PED volume and the presence of PED within the 1-mm central subfield, were selected as potential predictors for multivariate linear regression analysis using a stepwise forward selection procedure. We used *P* < 0.05 to retain predictors in the multivariate models. Residuals from the final models were evaluated to verify assumptions of normality and equal variance, and no adjustments were made for multiplicity.

## Results

A total of 249 patients were included in this analysis, with 126 eyes (50.6%) in the 3 + TAE group and 123 (49.4%) in the 3 + Q12W group. The baseline characteristics of the study population are summarized in Table 1. The median age of the patients was 64 (interquartile range, 59–68) years, and 95 of them were women (38.2%). The distribution of baseline characteristics between the two treatment groups is presented in Supplementary Table 1. None of the VIF values of independent variables exceeded 10, indicating negligible correlation among variables.

**Table 1:** Selected baseline characteristics for eyes included in this study

Characteristic	Total
Number of eyes	249
Sex, no. of females (%)	95 (38.2)
Age, median (IQR), years	64 (59–68)
Mean BCVA (SD), ETDRS letters	61.0 (14.3)
Baseline BCVA level, no. (%)	
Baseline BCVA < 34 letters	11 (4.4)
34 letters ≤ Baseline BCVA ≤ 73 letters	189 (75.9)
Baseline BCVA < 73 letters	49 (19.7)
Mean CRT (SD), μm	416.9 (130.7)
CRT level, no. (%)	
CRT ≤ 400 μm	91 (36.5)
CRT > 400 μm	158 (63.5)
Mean MRT (SD), μm	515.0 (155.4)
MRT level, no. (%)	
MRT ≤ 400 μm	65 (26.1)
MRT > 400 μm	184 (73.9)
Mean SFCT (SD), μm	266.4 (64.6)
Presence of IRF, no. (%)	170 (68.3)
Presence of SRF, no. (%)	233 (93.6)
SRF within 1-mm central subfield, no. (%)	156 (62.7)
Presence of PED, no. (%)	249 (100.0)
PED types, no. (%)	
I (fibrovascular PED)	198 (79.5)
II (hemorrhagic PED)	51 (20.5)
III (serous vascularized PED)	0 (0)
PED shapes, no. (%)	
Dome-shaped	57 (22.9)
Multilobular	77 (30.9)
Sharp peaked	72 (28.9)
Shallow irregular	36 (14.5)
Suspect RPE tear	7 (2.8)
PED within 1-mm central subfield, no. (%)	154 (61.8)
Mean PED maximum height (SD), μm	323.9 (183.9)
Mean PED volume (SD), mm^3^	0.69 (0.88)
Mean number of polypoidal lesions (SD), no.	3.2 (2.3)
Mean area of polypoidal lesions (SD), mm^2^	0.18 (0.19)
Polypoidal lesions within 1-mm central subfield, no. (%)^a^	100 (42.6)
Presence of BVN, no. (%)^a^	236 (94.8)
Mean area of BVN (SD), mm^2^	3.93 (4.01)
Presence of retinal hemorrhage, no. (%)	112 (45)
Mean area of retinal hemorrhage (SD), mm^2^	1.5 (3.9)
Retinal hemorrhage > 1 DD, no. (%)	27 (10.8)
Retinal hemorrhage within 1-mm central subfield, no. (%)	22 (8.8)
Presence of CVH, no. (%)^a^	169 (70.4)
CNV within 1 mm central subfield, no. (%)	14 (5.6)
GLD of total lesion (SD), μm	2720.5 (1227.6)

*BCVA* = best-corrected visual acuity; *BVN* = branching vascular network; *CNV* = choroidal neovascularization; *CRT* = central retinal thickness; *CVH* = choroidal vascular hyperpermeability; *DD* = disc diameter; *ETDRS* = Early Treatment Diabetic Retinopathy Study; *GLD* = greatest linear dimension; *IRF* = intraretinal fluid; *IQR* = interquartile range; *MRT* = maximum retinal thickness; *PED* = pigment epithelial detachment; *RPE* = retinal pigment epithelium; *SD* = standard deviation; *SFCT* = subfoveal choroidal thickness; *SRF* = subretinal fluid

^a^Eyes with undefined values had polypoidal lesions within 1-mm central subfield (n = 5), presence of BVN (n = 10), or presence of CVH (n = 9)

### Relationship between baseline characteristics and change in BCVA from baseline to week 48

There was no significant difference in baseline BCVA (EDTR letters) between the 3 + Q12W and 3 + TAE groups (61.5 ± 14.0 vs. 60.4 ± 14.6 letters, *P* = 0.53; Supplementary Table 1). At week 48, both groups demonstrated an improvement in the mean BCVA change from baseline, regardless of the treatment regimen (5.18 ± 16.24 vs. 6.29 ± 13.23 letters, *P* = 0.56). Table 2 outlines the results of univariate analysis of BCVA change at week 48, highlighting candidate predictors along with their coefficients (B) and *P* values. The factors associated with BCVA gain at week 48 included age, baseline BCVA, CRT, MRT, PED within the 1-mm central subfield, maximum PED height, PED volume, PED types, PED morphology, number of polypoidal lesions, area of polypoidal lesions, presence of CVH, BVN within the 1-mm central subfield, and polypoidal lesions within the same subfield.

**Table 2:** The correlation between baseline characteristics and changes in BCVA, CRT or MRT at week 48 using univariate analysis

Baseline characteristics	Change in BCVA from baseline to week 48		Change in CRT from baseline to week 48		Change in MRT from baseline to week 48	
	Unstandardized coefficients B (95% CI)	*P* value	Unstandardized coefficients B (95% CI)	*P* value	Unstandardized coefficients B (95% CI)	*P* value
Sex (women = 0)	1.08 (− 2.73, 4.88)	0.58	5.88 (− 32.02, 43.77)	0.76	8.73 (− 39.58, 57.04)	0.72
Age	− 0.24 (− 0.49, 0.02)	0.07	0.12 (− 2.44, 2.68)	0.93	− 0.15 (− 3.41, 3.12)	0.93
Treatment regimen (3 + TAE = 0 and 3 + Q12W = 1)	− 1.11 (− 4.80, 2.59)	0.56	10.27 (− 26.54, 47.07)	0.58	20.86 (− 26.02, 67.73)	0.38
BCVA (ETDRS letters)	− 0.45 (− 0.57, − 0.33)	< 0.01	2.07 (0.80, 3.34)	< 0.01	2.58 (0.96, 4.20)	< 0.01
BCVA < 34	18.97 (9.64, 28.30)	< 0.01	− 122.16 (− 217.89, − 26.44)	0.01	− 140.19 (− 262.50, 17.89)	0.03
34 ≤ BCVA ≤ 73	8.93 (4.45, 13.41)	< 0.01	− 43.13 (− 89.12, 2.87)	0.07	− 54.51 (− 113.27, 4.26)	0.07
BCVA > 73	[Reference]		[Reference]		[Reference]	
CRT (μm)	− 0.01 (− 0.02, 0.00)	0.15	− 0.58 (− 0.70, − 0.46)	< 0.01	− 0.57 (− 0.41, − 0.74)	< 0.01
CRT > 400 μm	− 4.52 (− 8.32, − 0.72)	0.02	− 160.71 (− 142.62, − 70.80)	< 0.01	− 97.75 (− 145.08, − 50.43)	< 0.01
CRT ≤ 400 μm	[Reference]		[Reference]		[Reference]	
MRT (μm)	− 0.01 (− 0.02, 0.00)	0.12	− 0.43 (− 0.53, − 0.32)	< 0.01	− 0.55 (− 0.68, − 0.41)	< 0.01
MRT > 400 μm	− 2.06 (− 6.86, 2.74)	0.40	− 4.30 (− 49.74, 41.14)	0.85	− 18.30 (− 77.93, 41.32)	0.55
MRT ≤ 400 μm	[Reference]		[Reference]		[Reference]	
SFCT (μm)	− 0.01 (− 0.04, 0.02)	0.47	0.01 (− 0.30, 0.32)	0.96	0.05 (− 0.34, 0.44)	0.79
Presence of IRF^b^	1.49 (− 2.49, 5.48)	0.46	− 43.53 (− 82.93, − 4.12)	0.03	− 47.98 (− 98.37, 2.41)	0.06
Presence of SRF^b^	− 7.29 (− 14.56, − 0.02)	0.05	− 63.39 (− 135.95, 9.17)	0.09	− 5.94 (− 99.00, 87.13)	0.90
SRF within 1-mm central subfield^b^	− 1.98 (− 5.80, 1.85)	0.31	− 17.93 (− 56.06, 20.20)	0.36	27.33 (− 21.19, 75.84)	0.27
PED within 1-mm central subfield^b^	− 1.46 (− 5.30, 2.38)	0.46	8.68 (− 29.53, 46.90)	0.66	8.57 (− 40.16, 57.29)	0.73
PED maximum height (μm)	− 0.02 (− 0.03, − 0.01)	< 0.01	0.02 (− 0.08, 0.13)	0.68	− 0.03 (− 0.16, 0.11)	0.70
PED volume (mm^3^)	− 2.21 (− 4.39, − 0.03)	0.05	6.69 (− 15.00, 28.38)	0.54	− 4.98 (− 32.49, 22.54)	0.72
PED types						
I (fibrovascular PED)	[Reference]		[Reference]		[Reference]	
II (hemorrhagic PED)	− 3.37 (− 7.93, 1.19)	0.15	− 53.01 (− 98.22, − 7.96)	0.02	− 97.6 (− 154.44, − 40.72)	< 0.01
III (serous vascularized PED)	none	–	none	–	none	–
PED shapes						
Dome-shaped	[Reference]		[Reference]		[Reference]	
Multilobular	1.30 (− 3.74, 6.34)	0.61	48.43 (− 1.91, 98.76)	0.06	82.78 (18.87, 146.68)	0.01
Sharp peaked	5.33 (0.22, 10.44)	0.04	8.39 (− 42.69, 59.47)	0.75	36.48 (− 28.36, 101.32)	0.27
Shallow irregular	5.43 (− 0.71, 11.57)	0.08	− 17.82 (− 79.16, 43.52)	0.57	6.55 (− 71.31, 84.41)	0.87
Suspect RPE tear	11.60 (0.05, 23.15)	0.05	− 26.57 (− 141.97, 88.82)	0.65	− 47.75 (− 194.23, 98.73)	0.52
Polypoidal lesions number (no.)	− 0.73 (− 1.52, 0.06)	0.07	1.57 (− 6.25, 9.38)	0.69	3.31 (− 6.82, 13.44)	0.52
Polypoidal lesions area (mm^2^)	− 13.21 (− 22.78, − 3.64)	< 0.01	23.48 (− 71.52, 118.48)	0.63	39.70 (− 83.38, 162.78)	0.53
Polypoidal lesions within 1-mm central subfield^b^	− 3.50 (− 7.27, 0.26)	0.07	4.23 (− 33.00, 41.45)	0.82	2.31 (− 45.97, 50.59)	0.93
Presence of BVN^b^	− 5.64 (− 22.02, 10.73)	0.50	54.70 (− 108.89, 218.28)	0.51	66.28 (− 148.55, 281.11)	0.54
BVN area (mm^2^)	− 0.03 (− 0.49, 0.43)	0.89	3.75 (− 0.80, 8.30)	0.11	3.20 (− 2.80, 9.19)	0.30
Presence of retinal hemorrhage^b^	− 1.94 (− 5.97, 2.10)	0.35	− 25.89 (− 66.04, 14.26)	0.21	− 24.77 (− 76.04, 26.49)	0.34
Retinal hemorrhage area (mm^2^)	− 0.10 (− 0.58, 0.38)	0.69	− 1.98 (− 6.79, 2.83)	0.42	− 1.47 (− 7.62, 4.68)	0.64
Retinal hemorrhage > 1 DD^b^	− 2.37 (− 7.13, 2.38)	0.33	− 23.35 (− 70.70, 24.00)	0.33	− 21.46 (-81.89, 38.96)	0.49
Retinal hemorrhage within 1-mm central subfield^b^	− 0.71 (− 7.22, 5.80)	0.83	16.77 (− 48.06, 81.60)	0.61	61.26 (− 21.09, 143.61)	0.14
Presence of CVH^b^	2.81 (− 1.46, 7.06)	0.19	4.60 (− 37.72, 46.92)	0.83	2.90 (− 51.00, 56.80)	0.92
CNV within 1-mm central subfield^b^	6.66 (− 1.33, 14.65)	0.10	− 65.75 (− 145.52, 14.01)	0.11	− 59.47 (− 161.51, 42.58)	0.25
GLD (μm)	0.00 (− 0.01, 0.01)	0.51	0.01 (− 0.01, 0.02)	0.23	0.01 (− 0.01, 0.03)	0.48

*BCVA* = best-corrected visual acuity; *BVN* = branching vascular network; *CI* = confidence interval; *CNV* = choroidal neovascularization; *CRT* = central retinal thickness; *CVH* = choroidal vascular hyperpermeability; *DD* = disc diameter; *ETDRS* = Early Treatment Diabetic Retinopathy Study; *GLD* = greatest linear dimension; *IRF* = intraretinal fluid; *MRT* = maximum retinal thickness; *PED* = pigment epithelial detachment; *RPE* = retinal pigment epithelium; *SFCT* = subfoveal choroidal thickness; *SRF* = subretinal fluid

^b^In univariate regression analysis, we used "0" to indicate absence and "1" to indicate presence

**Fig. 1:**
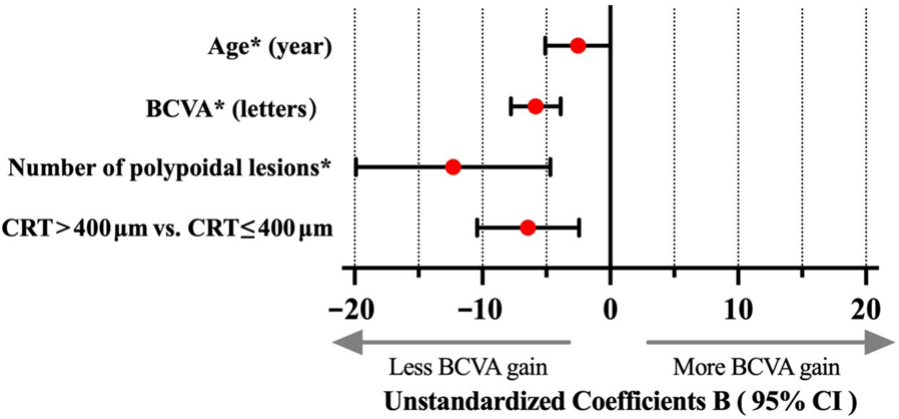
Forest plots of factors affecting the change in BCVA from baseline to week 48. Multivariate linear regression analysis was used to analyze potential factors influencing the change in BCVA from baseline to week 48. Eyes with younger age (95% CI, − 5.1 to 0.0; *P* = 0.04), worse baseline BCVA (95% CI, − 7.8 to − 3.9; *P* < 0.01), and fewer polypoidal lesions (95% CI, − 20.0 to − 5.0; *P* < 0.01) at baseline exhibited greater BCVA gains at week 48. Additionally, compared with eyes with CRT ≤ 400 μm at baseline, those with CRT > 400 μm showed less BCVA gain from baseline to week 48 (95% CI, − 10.8 to − 3.0; *P* < 0.01). *Coefficients B of age, BCVA and number of polypoidal lesions is expressed as 10 times the original value. BCVA, best-corrected visual acuity; CRT, central retinal thickness; CI, confidence interval

In the final multivariate model, four baseline factors–BCVA, CRT, number of polypoidal lesions, and age–were significantly associated with BCVA gain at week 48 (Fig. 1 and Table 3). A worse baseline BCVA (*P* < 0.01), fewer polypoidal lesions (*P* < 0.01), and younger age (*P* = 0.04) were correlated with greater BCVA gain at week 48. Additionally, eyes with CRT ≤ 400 μm at baseline experienced a greater BCVA gain at week 48 than those with CRT > 400 μm (*P* < 0.01).

**Table 3:** Multivariable analysis of baseline characteristics associated with BCVA, CRT and MRT changes

Baseline characteristics	Number of eyes	Unstandardized coefficients B (95% CI)	*P* value
*Change in BCVA from baseline to week 48*			
BCVA	249	− 0.58 (− 0.78, − 0.39)	< 0.01
CRT level			
CRT > 400 μm vs. CRT ≤ 400 μm	249	− 6.92 (− 10.81, − 3.03)	< 0.01
Number of polypoidal lesions	249	− 1.23 (− 1.99, − 0.74)	< 0.01
Age	249	− 0.25 (− 0.51, 0.00)	0.04
*Change in CRT from baseline to week 48*			
BCVA level (ETDRS letters)			
BCVA < 34 vs. BCVA > 73	60	− 107.03 (− 183.77, − 30.28)	0.01
34 ≤ BCVA ≤ 73 vs. BCVA > 73	238	− 46.15 (− 84.57, − 7.74)	0.02
CRT	249	− 0.70 (− 0.87, − 0.53)	< 0.01
CRT level			
CRT > 400 μm vs. CRT ≤ 400 μm	249	55.86 (6.50, 105.22)	0.03
PED volume	49	30.95 (10.50, 51.40)	< 0.01
PED types			
Hemorrhagic PED vs. Fibrovascular PED	249	− 61.42 (− 113.62, − 9.21)	0.02
PED shapes			
Shallow irregular vs. Multilobular	113	− 84.32 (− 130.81, − 37.83)	< 0.01
Sharp peaked vs. Multilobular	149	− 60.15 (− 99.83, − 20.49)	< 0.01
*Change in MRT from baseline to week 48*			
BCVA level (ETDRS letters)			
BCVA < 34 vs. BCVA > 73	60	− 133.11 (− 241.32, 33.39)	0.02
MRT	249	− 0.79 (− 1.00, − 0.58)	< 0.01
CRT level			
CRT > 400 μm vs. CRT ≤ 400 μm	249	89.20 (26.97, 151.43)	0.01
PED volume	249	45.49 (16.45, 74.54)	< 0.01
PED types			
Hemorrhagic PED vs. Fibrovascular PED	249	− 78.05 (− 150.11, − 5.99)	0.03
PED shapes			
Shallow irregular vs. Multilobular	113	− 108.57 (− 174.24, − 42.90)	< 0.01
Sharp peaked vs. Multilobular	149	− 79.52 (− 135.02, − 24.01)	0.01

*BCVA* = best-corrected visual acuity; *CI* = confidence interval; *CRT* = central retinal thickness; *ETDRS* = Early Treatment Diabetic Retinopathy Study; *MRT* = maximum retinal thickness; *PED* = pigment epithelial detachment

For every 10-letter improvement in baseline BCVA, the BCVA gain at week 48 decreased by 5.8 letters [95% confidence interval (CI), − 7.8 to − 3.9 letters]. Furthermore, at week 48, the BCVA gain was 6.9 letters less for eyes with severe edema (CRT > 400 μm) than for those with mild edema (CRT ≤ 400 μm) (95% CI, − 10.8 to − 3.0 letters). Additionally, for each increase of one polypoidal lesion at baseline, the BCVA gain decreased by 1.2 letters (95% CI, − 2.0 to − 0.5 letters) at week 48. For every 10-year increase in age at baseline, the BCVA gain at week 48 reduced by 2.5 letters (95% CI, − 5.1 to 0.0 letters).

No significant interactions were noted between these factors and treatment regimen (Supplementary Table 2). Based on the final model, the adjusted R^2^ value for the change in BCVA from baseline to week 48 was 0.28.

### Relationship between baseline characteristics and change in CRT from baseline to week 48

There was no significant difference in baseline CRT between the 3 + Q12W and 3 + TAE groups (422.9 ± 135.4 vs. 411.1 ± 126.3 μm, *P* = 0.55; Supplementary Table 1). The univariate analysis presented in Table 2 revealed several candidate factors associated with the change in CRT at week 48, including baseline BCVA, CRT, MRT, presence of IRF, presence of SRF, PED within the 1-mm central subfield, PED volume, PED types, PED morphology, BVN area, and CNV within the 1-mm central subfield.

**Fig. 2:**
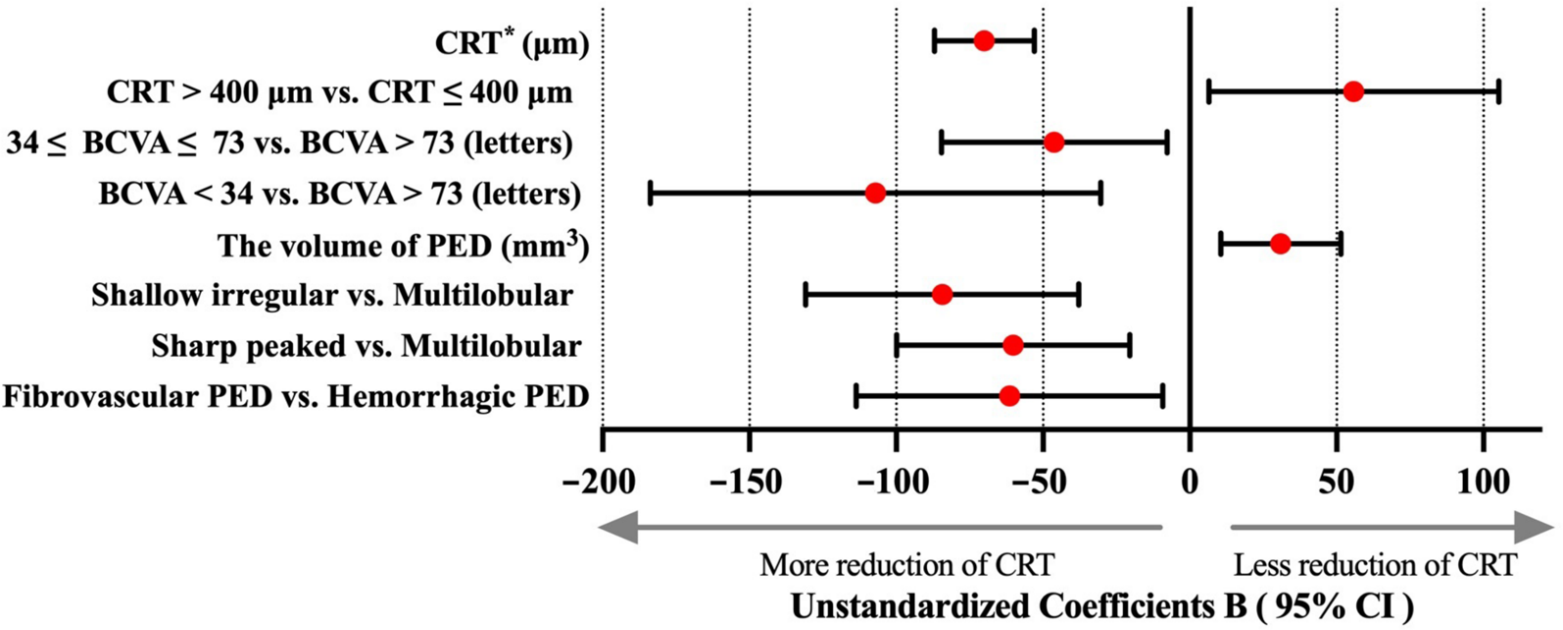
Forest plots of factors affecting the change in CRT from baseline to week 48. Multivariate linear regression analysis was used to analyze potential factors influencing the change in CRT from baseline to week 48. Eyes with higher CRT (95% CI, − 0.9 to − 0.5; *P* < 0.01) and smaller PED volume (95% CI, 10.5 to 51.4; *P* < 0.01) at baseline achieved greater CRT reductions at week 48. Compared with eyes with baseline BCVA > 73 letters, those with baseline BCVA < 34 letters and those with baseline BCVA of 34 to 73 letters exhibited greater CRT reductions from baseline to week 48 (95% CI, − 183.8 to − 30.1 and − 84.6 to − 7.7; *P* = 0.01 and *P* = 0.02, respectively). Compared with eyes having CRT ≤ 400 μm at baseline, those with CRT > 400 μm exhibited a lower reduction in CRT at week 48 (95% CI, 6.5 to 105.2; *P* = 0.03). Eyes with hemorrhagic PEDs demonstrated greater reductions in CRT than those with fibrovascular PEDs at week 48 (95% CI, − 113.6 to − 9.2; *P* = 0.02). Additionally, compared with eyes with multilobular PED, those with shallow irregular or sharp-peaked PEDs exhibited greater reductions in CRT from baseline to week 48 (95% CI, − 130.8 to − 37.8 and − 99.8 to − 20.5; both *P* < 0.01). * Coefficients B of CRT is expressed as 100 times the original value. BCVA, best-corrected visual acuity; CRT, central retinal thickness; PED, pigment epithelial detachment; CI, confidence interval

In the final multivariate model, six baseline factors–BCVA level, CRT, CRT level, PED volume, PED types, and PED morphology–were significantly associated with CRT reduction at week 48 (Fig. 2 and Table 3). Higher CRT (*P* < 0.01) and smaller PED volume (*P* < 0.01) were associated with a more pronounced reduction in CRT from baseline to week 48. Additionally, compared with eyes with severe (< 34 letters) and moderate (34–73 letters) visual impairment at baseline, those with relatively good BCVA (> 73 letters) exhibited a lower reduction in CRT from baseline to week 48 (*P* = 0.01 and *P* = 0.02, respectively). Eyes with hemorrhagic PEDs exhibited a greater reduction in CRT at week 48 than those with fibrovascular PEDs (*P* = 0.02). Furthermore, eyes with shallow irregular or sharp-peaked PEDs exhibited greater reductions in CRT from baseline to week 48 than those with multilobular PEDs (both *P* < 0.01).

For every 10-μm increase in baseline CRT, there was a corresponding reduction in CRT of 7.0 μm (95% CI, 5.3–8.7 μm) at week 48. However, for eyes with baseline CRT > 400 μm, the reduction in CRT was 55.9 μm (95% CI, 6.5–105.2 μm) lower at week 48. Additionally, for every 1-mm^3^ increase in PED volume at baseline, the reduction in CRT was 31.0 μm (95% CI, 10.5–51.4 μm) lower at week 48. Eyes with relatively good BCVA (> 73 letters) at baseline had 107.0 and 46.2 μm lower reduction in CRT at week 48 than those with severe and moderate visual impairment, respectively (95% CI, 30.3–183.8 μm and 7.8–84.6 μm). Compared with eyes with multilobular PEDs, those with shallow irregular or sharp-peaked PEDs showed 60.2 μm (95% CI, 20.5–99.8 μm) and 84.3 μm (95% CI, 37.8–130.8 μm) greater reductions in CRT from baseline to week 48.

No significant interactions were observed between these factors and treatment regimen (Supplementary Table 3). Based on the final model, the adjusted R^2^ value for the change in CRT from baseline to week 48 was 0.40.

### Relationship between baseline characteristics and change in MRT from baseline to week 48

There was no significant difference in baseline MRT between the 3 + Q12W and 3 + TAE groups (518.8 ± 162.3 vs. 511.3 ± 149.0 μm, *P* = 0.71; Supplementary Table 1). The univariate analysis presented in Table 2 revealed several candidate factors associated with the change in MRT at week 48, including baseline BCVA, CRT, MRT, presence of IRF, presence of PED, PED within the 1-mm central subfield, PED volume, PED types, PED morphology, and retinal hemorrhage within the 1-mm central subfield.

**Fig. 3:**
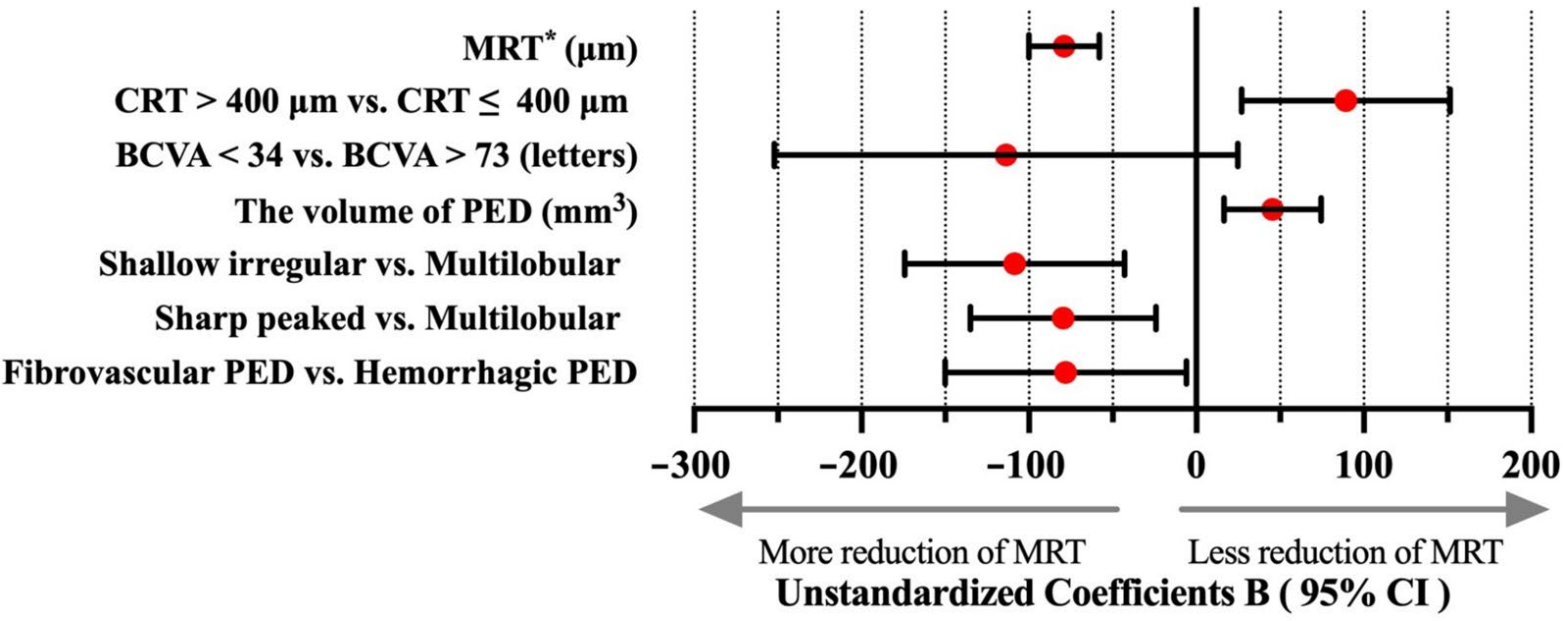
Forest plots of factors affecting the change in MRT from baseline to week 48. Multivariate linear regression analysis was used to analyze potential factors influencing the change in MRT from baseline to week 48. Eyes with higher MRT (95% CI, − 1.0 to − 0.6; *P* < 0.01) and smaller PED volume (95% CI, 16.45 to 74.54; *P* < 0.01) at baseline showed greater MRT reductions at week 48. Compared with eyes with CRT ≤ 400 μm at baseline, those with CRT > 400 μm (95% CI, 27.0 to 151.4; *P* = 0.01) showed a lower reduction in MRT at week 48. Compared with eyes of baseline BCVA > 73 letters, those of baseline BCVA < 34 letters exhibited greater reductions in MRT from baseline to week 48 (95% CI, − 241.3 to 33.4; *P* = 0.02). Eyes with hemorrhagic PEDs exhibited greater reductions in MRT than those with fibrovascular PEDs at week 48 (95% CI, − 150.1 to − 6.0; *P* = 0.03). Additionally, compared with eyes with multilobular PEDs, those with shallow irregular or sharp-peaked PEDs exhibited greater reductions in MRT from baseline to week 48 (95% CI, − 174.2 to − 42.9 and − 135.0 to − 24.0; *P* < 0.01 and *P* = 0.01, respectively). * Coefficients B of MRT is expressed as 100 times the original value. BCVA, best-corrected visual acuity; CRT, central retinal thickness; MRT, maximum retinal thickness; PED, pigment epithelial detachment; CI, confidence interval

In the final multivariate model, six baseline characteristics–BCVA level, MRT, CRT level, PED volume, PED types, and PED morphology–were significantly associated with MRT reduction at week 48 (Fig. 3 and Table 3). Higher MRT (*P* < 0.01) and smaller PED volume (*P* < 0.01) were associated with a more pronounced reduction in MRT from baseline to week 48. Compared with eyes with severe visual impairment (< 34 letters) at baseline, those with relatively good BCVA (> 73 letters) showed a lower reduction in MRT (*P* = 0.02). Eyes with hemorrhagic PEDs demonstrated a greater reduction in MRT at week 48 than those with fibrovascular PEDs (*P* = 0.03). Furthermore, eyes with shallow irregular or sharp-peaked PEDs exhibited a greater reduction in MRT from baseline to week 48 than those with multilobular PEDs (*P* = 0.01 and *P* < 0.01, respectively).

For every 10-μm increase in baseline MRT, the reduction in MRT at week 48 was 7.9 μm (95% CI, 5.8–10.0 μm) greater. Eyes with baseline CRT > 400 μm showed a 89.2 μm (95% CI, 27.0–151.4 μm) lower reduction in MRT at week 48. Additionally, for every 1-mm^3^ increase in PED volume at baseline, the reduction in MRT was 45.5 μm (95% CI, 16.5–74.5 μm) lower at week 48. Compared with eyes with severe visual impairment (< 34 letters), those with relatively good BCVA (> 73 letters) at baseline showed 133.1 μm (95% CI, − 241.3–33.4 μm) lower reduction in MRT at week 48. Furthermore, compared with eyes with multilobular PEDs, those with shallow irregular or sharp-peaked PEDs exhibited 108.6 μm (95% CI, 42.9–174.2 μm) and 79.5 μm (95% CI, 24.0–135.0 μm) greater reductions in MRT at week 48.

No significant interactions were observed between these factors and treatment regimens (Supplementary Table 4). Based on the final model, the adjusted R^2^ value for the change in MRT from baseline to week 48 was 0.30.

## Discussion

This exploratory post hoc analysis provided additional insights into potential predictors of visual and anatomical outcomes in patients with PCV receiving IVI of conbercept.

Baseline visual acuity (VA) has consistently been identified as a key factor influencing visual outcomes in anti-VEGF therapy for nAMD across numerous studies [10, 21, 28–30]. As eyes with better baseline BCVA may experience limited visual gain due to the ceiling effect, many clinical trials exclude patients with relatively good BCVA. For example, the EVEREST study [13], which compared PDT combined with ranibizumab with ranibizumab monotherapy for PCV, excluded patients with baseline BCVA exceeding 78 letters. Similarly, the LAPTOP [12] and PLANET [29] trials excluded eyes with baseline BCVA better than 71 and 73 letters, respectively. In the STAR study, however, 20% (49/249) of the enrolled patients had a baseline BCVA exceeding 73 letters, and this subgroup experienced a mean BCVA decrease of 1.88 letters at week 48. Our post hoc analysis revealed that for every 10-letter improvement in baseline BCVA, the BCVA gain at week 48 was 5.8 letters less. In addition to the ceiling effect, a possible explanation is the higher proportion of patients requiring rescue PDT among patients with baseline BCVA exceeding 73 letters than among those with lower BCVA (12.2% vs. 8.5%; Chi-squared test, *P* = 0.417). In the STAR study, six eyes receiving rescue PDT with baseline BCVA exceeding 73 letters showed a mean decrease of 7.4 letters after the administration of the loading dose, deteriorating to 15.0 letters at week 24 and eventually stabilizing at 14 letters at week 48. This raised concerns that PCV eyes with BCVA exceeding 73 letters may experience significant visual loss during the loading phase of anti-VEGF monotherapy and that BCVA may not improve even after rescue PDT.

MRT has rarely been explored in clinical trials of nAMD. However, considering that PCV has distinct characteristics compared with typical type 1 MNV, we included this parameter in our analysis. In the STAR study, PED within the 1-mm central subfield was observed in only 61.8% of the eyes. When the center of the PED does not overlap with the foveal center, MRT serves as an additional parameter alongside CRT to provide a more comprehensive view of macular elevation. In our study, we found that higher baseline MRT, rather than CRT, was associated with a greater reduction in MRT at week 48 following treatment with conbercept for PCV. This supports the notion that MRT may be a more critical predictor of anatomical changes after anti-VEGF therapy for PCV.

The influence of PED on the visual and anatomical outcomes of anti-VEGF therapy in nAMD has been reported inconsistently. Although most studies have indicated that the presence of PED is associated with a slight reduction in visual gains during the follow-up period, there is no clear evidence that the presence of a PED or its morphological parameters—such as height, area, and volume—significantly impact visual outcomes [30–33]. Additionally, data regarding the effect of PED on the outcomes of PCV treatment are limited, and these findings are often conflicting. Kim et al. found that the presence of subfoveal fibrovascular PED was associated with a high incidence of lesion reactivation and poor outcomes in eyes with age-related macular degeneration and PCV [32]. Another study indicated that fibrovascular PED at baseline was associated with a higher recurrence rate of nAMD following anti-VEGF therapy [34]. In this post hoc study, we investigated the effects of PED type and morphology on the outcomes of anti-VEGF therapy in PCV. We found that the volume, type, and morphology of PEDs significantly affected changes in CRT and MRT from baseline to 48 weeks but did not have a significant impact on visual outcomes at week 48. Specifically, eyes with hemorrhagic PEDs exhibited a greater reduction in CRT and MRT at week 48 than those with fibrovascular PEDs. Additionally, eyes with shallow irregular PED or sharp-peaked PED showed more significant reductions in CRT and MRT at week 48 than those with multilobular PED.

PEDs are reportedly observed in 30% to 80% of patients with nAMD; however, they were observed in all patients in the current study [31, 32, 35–39]. One reason for this discrepancy is that definitions of PED vary among studies, with some specifying minimum height or width on OCT and others lacking specific criteria. Additionally, advancements in OCT resolution have made it easier to identify RPE elevation, increasing the prevalence of PEDs, particularly shallow PEDs, in eyes with PCV. In the STAR study, the mean baseline PED height was 323.9 μm, which decreased by 105.0 μm in the 3 + Q12W group and 71.8 μm in the 3 + TAE group [24]. Although the univariate analysis indicated that PED height was related to changes in BCVA at week 48, the coefficient B was close to zero, preventing it from being included in the final multivariate model. The mean baseline PED volume in the STAR study was 0.69 mm^3^, which decreased by 16.6% in the 3 + Q12W group and 3.3% in the 3 + TAE group at week 48 [24]. In this post hoc analysis, we found that smaller PED volume was associated with greater reductions in CRT and MRT.

The characteristics of polypoidal lesions, such as fewer lesions, smaller area, and limited progression, have been associated with higher regression rates following anti-VEGF therapy [40, 41]. In our study, we found that a small number of polypoidal lesions were associated with greater BCVA gain. A possible explanation is that fewer polypoidal lesions reduce the risk of rupture or retinal hemorrhage, which can lead to severe visual loss. A study investigating the relationship between polypoidal lesions and PCV recurrence demonstrated that an increase in the area of polypoidal lesions indicated a higher likelihood of recurrence [42]. However, further study is needed to clarify the relationship between changes in the number of polypoidal lesions and the prognosis of anti-VEGF therapy for PCV.

The strengths of this study include a relatively large sample size, data collected from RCTs, and an investigation of the effect of PED type and morphology on the visual and anatomical outcomes of anti-VEGF therapy in PCV. However, there are several limitations. First, the post hoc nature of the study may introduce biases. False discovery rate (FDR) adjustment was not applied in this exploratory study to mitigate the risk of type II errors, thereby preserving potentially meaningful findings for future investigation. However, the absence of multiple testing correction may increase the likelihood of type I errors; thus, the unadjusted results should be interpreted with caution and require further validation. Second, the study exclusively enrolled a Chinese population, limiting the ability to generalize findings across different racial groups. Third, some outcomes related to VA, such as RPE tear, macular atrophy, and fibrosis, were not analyzed. Fourth, BVN and polypoidal lesion area measurement may not be accurate in all cases, especially when retinal hemorrhage is severe and blocked the hyperfluorescent of BVN and polyps in ICGA. Furthermore, measurements can be different when they were evaluated in OCTA imaging. Despite these limitations, our study provides valuable insights into predictors associated with visual and anatomical outcomes of conbercept treatment for PCV. Notably, these outcomes remain positive and hold clinical significance, even in the presence of factors associated with a less optimal response.

## Conclusions

Our study identified the baseline factors associated with the outcomes of conbercept treatment for active PCV in a Chinese population. Greater gains in BCVA were associated with worse baseline BCVA, CRT ≤ 400 μm, fewer polypoidal lesions, and younger age at baseline. Conversely, higher CRT and MRT, smaller PED volume, baseline BCVA ≤ 73 letters, hemorrhagic PED, and shallow irregular or sharp-peaked PEDs were associated with a greater reduction in CRT and MRT at week 48.

## Supplementary Information

Additional file1 (DOCX 52 KB)

## Abbreviations

PCV: Polypoidal choroidal vasculopathy
BCVA: Best-corrected visual acuity
CRT: Central retinal thickness
MRT: Maximum retinal thickness
PED: Pigment epithelial detachment
IVI: Intravitreal injection
PDT: Photodynamic therapy
nAMD: Neovascular age-related macular degeneration
CNV: Choroidal neovascularization
OCT: Optical coherence tomography
CFP: Color fundus photography
ICGA: Indocyanine green angiography
GLD: Greatest linear dimension
IRF: Intraretinal fluid
ILM: Internal limiting membrane
SFCT: Subfoveal choroidal thickness
CVH: Choroidal vascular hyperpermeability

## Declarations

### Availability of data and materials

The data that support the findings of this study are available from the corresponding author upon reasonable request.
