# Supplementary material for: Prognostic factors in the treatment of polypoidal choroidal vasculopathy with conbercept: a post hoc analysis of the STAR study
Source: Eye Vis (Lond). 2025 Jun 18;12:24. doi: 10.1186/s40662-025-00441-5 (PMC12175423; doi:10.1186/s40662-025-00441-5)
Supplement: Supplementary file 1 — Additional file1 (DOCX 52 KB) [file 40662_2025_441_MOESM1_ESM.docx]

**Supplementary Table 1.** Selected baseline characteristics for study eyes that completed 48-week visit by treatment group

| Characteristic | 3+Q12W | 3+TAE | *P* value | All |
| --- | --- | --- | --- | --- |
| No. of eyes | 123 (49.4) | 126 (50.6) |  | 249 |
| Gender, women, no. (%) | 44 (35.8) | 51 (40.5) | 0.45 | 95 (38.2) |
| Age, median (IQR) | 64.1 (60-68) | 64.0 (59-68) | 0.95 | 64 (59-68) |
| Mean BCVA (SD), ETDRS letters | 61.5 (14.0) | 60.4 (14.6) | 0.53 | 61.0 (14.3) |
| Mean CRT, μm (SD) | 422.9 (135.4) | 411.1 (126.3) | 0.55 | 416.9 (130.7) |
| Mean MRT, μm (SD) | 518.8 (162.3) | 511.3 (149.0) | 0.71 | 515.0 (155.4) |
| Mean SFCT, μm (SD) | 265.3 (67.5) | 267.5 (61.9) | 0.80 | 266.4 (64.6) |
| Presence of IRF, no. (%) | 85 (69.1) | 85 (67.5) | 0.78 | 170 (68.3) |
| Presence of SRF, no. (%) | 119 (96.7) | 114 (90.5) | 0.04 | 233 (93.6) |
| SRF within 1-mm central subfield, no. (%) | 80 (65.0) | 76 (60.3) | 0.49 | 156 (62.7) |
| Presence of PED, no. (%) | 123 (100.0) | 126 (100.0) | >0.99 | 249 (100.0) |
| PED types, no. (%)  I (Fibrovascular PED)  II (Hemorrhagic PED)  III (Serous vascularized PED) | 92 (74.8)  31 (25.2)  0 | 106 (84.1)  20 (15.9)  0 | 0.08 | 249  198 (79.5)  51 (20.5)  0 (0.0) |
| PED shapes, no. (%)  Dome-shaped  Multilobular  Sharp peaked  Shallow irregular  Suspect RPE tear | 30 (24.4)  37 (30.1)  30 (24.4)  21 (17.1)  5 (4.0) | 27 (21.4)  40 (31.8)  42 (33.3)  15 (11.9)  2 (1.6) | 0.34 | 249  57 (22.9)  77 (30.9)  72 (28.9)  36 (14.5)  7 (2.8) |
| PED within 1-mm central subfield, no. (%) | 80 (65.0) | 74 (58.7) | 0.38 | 154 (61.8) |
| Mean PED maximum height, μm (SD) | 343.3 (196.8) | 304.2 (168.3) | 0.15 | 323.9 (183.9) |
| Mean PED volume, mm^3^ (SD) | 0.78 (0.95) | 0.60 (0.79) | 0.26 | 0.69 (0.88) |
| Mean number of polypoidal lesions, no. (SD) | 3.1 (2.2) | 3.2 (2.4) | 0.71 | 3.2 (2.3) |
| Mean area of polypoidal lesions, mm^2^ (SD) | 0.19 (0.21) | 0.16 (0.17) | 0.33 | 0.18 (0.19) |
| Polypoidal lesions within 1-mm central subfield, no. (%)^a^ | 49 (42.6) | 51 (42.5) | 0.95 | 100 (42.6) |
| Presence of BVN, no. (%)^a^ | 115 (93.5) | 121 (96.0) | 0.60 | 236 (94.8) |
| Mean area of BVN, mm^2^ (SD) | 4.15 (4.70) | 3.72 (3.22) | 0.80 | 3.93 (4.01) |
| Presence of retinal hemorrhage, no. (%) | 64 (52.0) | 48 (38.1) | 0.03 | 112 (45) |
| Mean area of retinal hemorrhage, mm^2^ (SD) | 1.87 (4.61) | 1.14 (3.05) | 0.01 | 1.5 (3.9) |
| Retinal hemorrhage > 1 DD, no. (%) | 16 (13.0) | 11 (8.7) | 0.28 | 27 (10.8) |
| Retinal hemorrhage within 1-mm central subfield, no. (%) | 9 (7.3) | 13 (10.3) | 0.40 | 22 (8.8) |
| Presence of CVH, no. (%)^a^ | 79 (64.2) | 90 (71.4) | 0.11 | 169 (70.4) |
| CNV within 1-mm central subfield, no. (%) | 5 (4.1) | 9 (7.1) | 0.29 | 14 (5.6) |
| GLD of total lesion, μm (SD) | 2733.8 (1361.4) | 2707.8 (1092.5) | 0.89 | 2720.5 (1227.6) |

3+Q12W = 3 monthly injections followed by injections every 12 weeks; 3+TAE = 3 monthly injections followed by treat and extend regimen; BCVA = best-corrected visual acuity; BVN = branching vascular network; CNV = choroidal neovascularization; CRT = central retinal thickness; CVH = choroidal vascular hyperpermeability; DD = disk diameter; ETDRS = Early Treatment Diabetic Retinopathy Study; GLD = greatest linear dimension; IRF = intraretinal fluid; IQR = interquartile range; MRT = maximum retinal thickness; PED = pigment epithelial detachment; RPE = retinal pigment epithelium; SD = standard deviation; SFCT = subfoveal choroidal thickness; SRF = subretinal fluid.

^a^ Eyes with value of unable to define: presence of polypoidal lesions within 1-mm central subfield (n = 5), presence of BNN (n = 10), presence of CVH (n = 9).

**Supplementary Table 2.** Univariate analysis of the change in BCVA at week 48 for each candidate predictive factor by treatment group

| Change in BCVA from baseline at week 48 | 3+TAE | | 3+Q12W | | All | |
| --- | --- | --- | --- | --- | --- | --- |
|  | Unstandardized coefficients B  (95% CI) | *P* value | Unstandardized coefficients B  (95% CI) | *P* value | Unstandardized coefficients B  (95% CI) | *P* value |
| Gender (Women = 0) | 0.91 (−3.86, 5.68) | 0.71 | 1.38 (−4.69, 7.44) | 0.65 | 1.08(−2.73, 4.88) | 0.58 |
| Age | −0.18 (−0.50, 0.15) | 0.29 | −0.30 (−0.07, 0.10) | 0.07 | −0.24 (−0.49, 0.02) | 0.07 |
| Treatment regimen  (3+TAE = 0 and 3+Q12W = 1) |  |  |  |  | −1.11 (−4.80, 2.59) | 0.56 |
| BCVA (ETDRS letters) | −0.33 (−0.48, −0.18) | <0.01 | −0.58 (−0.76, −0.40) | <0.01 | −0.45 (−0.57, −0.33) | <0.01 |
| BCVA < 34 | 8.64 (−2.64, 19.91) | 0.13 | 34.02 (18.12, 49.92) | <0.01 | 18.97 (9.64, 28.30) | <0.01 |
| 34 ≤ BCVA ≤ 73 | 5.77 (−0.37, 11.91) | 0.07 | 11.49 (4.99, 17.98) | <0.01 | 8.93 (4.45, 13.41) | <0.01 |
| BCVA > 73 | [Reference] |  | [Reference] |  | [Reference] |  |
| CRT (μm) | 0.00 (−0.02, 0.01) | 0.80 | −0.02 (−0.03, 0.00) | 0.11 | −0.01 (−0.02, 0.00) | 0.15 |
| CRT > 400 | −1.25 (−6.18, 3.69) | 0.62 | −7.62 (−13.45, −1.80) | 0.01 | −4.52 (−8.32, −0.72) | 0.02 |
| CRT ≤ 400 | [Reference] |  | [Reference] |  | [Reference] |  |
| MRT (μm) | 0.00 (−0.02, 0.02) | 0.90 | −0.02 (−0.04, 0.00) | 0.07 | −0.01 (−0.02, 0.00) | 0.12 |
| MRT > 400 | −1.24 (−6.74, 4.25) | 0.66 | −2.91 (−9.41, 3.59) | 0.38 | −2.06 (−6.86, 2.74) | 0.40 |
| MRT ≤ 400 | [Reference] |  | [Reference] |  | [Reference] |  |
| SFCT (μm) | −0.03 (−0.07, 0.02) | 0.21 | 0.00 (−0.05, 0.05) | 0.97 | −0.01 (−0.04, 0.02) | 0.47 |
| Presence of IRF^b^ | 1.22 (−3.78, 6.22) | 0.63 | 1.84 (−4.51, 8.19) | 0.57 | 1.49 (−2.49, 5.48) | 0.46 |
| Presence of SRF^b^ | −6.20 (−13.82, 1.42) | 0.11 | −9.89 (−26.21, 6.42) | 0.23 | −7.29 (−14.56, −0.02) | 0.05 |
| SRF within 1-mm central subfield^b^ | −1.97 (−6.76, 2.83) | 0.42 | −1.91 (−8.00, 4.19) | 0.54 | −1.98 (−5.80, 1.85) | 0.31 |
| PED within 1-mm central subfield^b^ | −1.89 (−6.72, 2.95) | 0.44 | −0.87 (−6.97, 5.23) | 0.78 | −1.46 (−5.30, 2.38) | 0.46 |
| PED maximum height (μm) | −0.02 (−0.03, 0.00) | 0.05 | −0.02 (−0.03, 0.00) | 0.02 | −0.02 (−0.03, −0.01) | <0.01 |
| PED volume (mm^3^) | −2.08 (−5.23, 1.08) | 0.20 | −2.26 (−5.38, 0.86) | 0.15 | −2.21 (−4.39, −0.03) | 0.05 |
| PED types, no. (%)  I (Fibrovascular PED)  II (Hemorrhagic PED)  III (Serous vascularized PED) | [Reference]  −0.64 (−7,05, 5.77)  None | 0.84 | [Reference]  −5.16 (−11.80, 1.48)  None | 0.13 | [Reference]  −3.37 (−7.93, 1.19)  None | 0.15 |
| PED shapes, no. (%)  Dome-shaped  Multilobular  Sharp peaked  Shallow irregular  Suspect RPE tear | [Reference]  2.15 (−4.38, 8.68)  5.71 (−0.76, 12.18)  5.26 (3.18, 13.70)  −0.07 (−19.29, 19.14) | 0.52  0.08  0.22  0.99 | [Reference]  0.32 (−7.51, 8.14)  4.60 (−3.62, 12.82)  5.62 (−3.44, 14.68)  16.47 (1.09, 31.85) | 0.94  0.27  0.22  0.04 | [Reference]  1.30 (−3.74, 6.34)  5.33 (0.22, 10.44)  5.43 (−0.71, 11.57)  11.60 (0.05, 23.15) | 0.61  0.04  0.08  0.05 |
| Polypoidal lesions number (no.) | −0.95 (−1.91, 0.02) | 0.05 | −0.50 (−1.79, 0.80) | 0.45 | −0.73 (−1.52, 0.06) | 0.07 |
| Polypoidal lesions area (mm^2^) | −16.21 (−29.53, −2.87) | 0.02 | −10.79 (−24.81, 3.22) | 0.13 | −13.21 (−22.78, −3.64) | <0.01 |
| Polypoidal lesions within 1-mm central subfield^b^ | −1.03 (−5.81, 3.76) | 0.67 | −6.09 (−12.01, −0.18) | 0.04 | −3.50 (−7.27, 0.26) | 0.07 |
| Presence of BVN^b^ | −5.77 (−23.96, 12.41) | 0.53 | −5.04 (−36.43, 26.37) | 0.75 | −5.64 (−22.02, 10.73) | 0.50 |
| BVN area (mm^2^) | −0.46 (−1.18, 0.27) | 0.21 | 0.18 (−0.44, 0.80) | 0.56 | −0.03 (−0.49, 0.43) | 0.89 |
| Presence of retinal hemorrhage^b^ | 0.61 (−4.83, 6.04) | 0.83 | −3.85 (−9.92, 2.21) | 0.21 | −1.94 (−5.97, 2.10) | 0.35 |
| Retinal hemorrhage area (mm^2^) | 0.36 (−0.42, 1.13) | 0.37 | −0.28 (−0.92, 0.36) | 0.39 | −0.10 (−0.58, 0.38) | 0.69 |
| Retinal hemorrhage > 1 DD^b^ | 1.55 (−5.14, 8.24) | 0.65 | −4.95 (−11.83, 1.94) | 0.16 | −2.37 (−7.13, 2.38) | 0.33 |
| Retinal hemorrhage within 1-mm central subfield^b^ | −6.15 (−13.78, 1.47) | 0.11 | 6.64 (−4.47, 17.75) | 0.24 | −0.71 (−7.22, 5.80) | 0.83 |
| Presence of CVH^b^ | −2.90 (−8.66, 2.86) | 0.32 | 7.31 (1.06, 13.57) | 0.02 | 2.81 (−1.46, 7.06) | 0.19 |
| CNV within 1-mm central subfield^b^ | 8.83 (−0.16, 17.83) | 0.05 | 2.60 (−12.14, 17.33) | 0.73 | 6.66 (−1.33, 14.65) | 0.10 |
| GLD (μm) | 0.01 (−0.01, 0.00) | 0.07 | 0.00 (−0.01, 0.01) | 0.65 | 0.00 (−0.01, 0.01) | 0.51 |

3+Q12W = 3 monthly injections followed by injections every 12 weeks; 3+TAE = 3 monthly injections followed by treat and extend regimen; BCVA = best-corrected visual acuity; BVN = branching vascular network; CI = confidence interval; CNV = choroidal neovascularization; CRT = central retinal thickness; CVH = choroidal vascular hyperpermeability; DD = disk diameter; ETDRS = Early Treatment Diabetic Retinopathy Study; GLD = greatest linear dimension; IRF = intraretinal fluid; MRT = maximum retinal thickness; PED = pigment epithelial detachment; RPE = retinal pigment epithelium; SFCT = subfoveal choroidal thickness; SRF = subretinal fluid.

^b^ In unvariate regression analysis, we used "0" to indicate absence and "1" to indicate presence.

**Supplementary Table 3.** Univariate analysis of the change in CRT at week 48 for each candidate predictive factor by treatment group

| Change in CRT from baseline at week 48 | 3+TAE | | 3+Q12W | | All | |
| --- | --- | --- | --- | --- | --- | --- |
|  | Unstandardized coefficients B  (95% CI) | *P* value | Unstandardized coefficients B  (95% CI) | *P* value | Unstandardized coefficients B  (95% CI) | *P* value |
| Gender (Women = 0) | −8.64 (−60.52, 43.25) | 0.74 | 20.43 (−35.88, 76.74) | 0.47 | 5.88 (−32.02, 43.77) | 0.76 |
| Age | −1.18 (−4.72, 2.37) | 0.51 | 1.43 (−2.32, 5.18) | 0.45 | 0.12 (−2.44, 2.68) | 0.93 |
| Treatment regimen  (3+TAE = 0 and 3+Q12W = 1) |  |  |  |  | 10.27 (−26.54, 47.07) | 0.58 |
| BCVA (ETDRS letters) | 1.99 (0.27, 3.70) | 0.02 | 2.14 (0.23, 4.05) | 0.03 | 2.07 (0.80, 3.34) | <0.01 |
| BCVA < 34 | −147.42 (−269.08, −25.76) | 0.02 | −82.76 (−243.06, 77.54) | 0.31 | −122.16 (−217.89, −26.44) | 0.01 |
| 34 ≤ BCVA ≤ 73 | −52.15 (−118.35, 14.06) | 0.12 | −34.85 (−100.33, 30.64) | 0.29 | −43.13 (−89.12, 2.87) | 0.07 |
| BCVA > 73 | [Reference] |  | [Reference] |  | [Reference] |  |
| CRT (μm) | −0.69 (−0.82, −0.55) | <0.01 | −0.35 (−0.51, −0.18) | <0.01 | −0.58 (−0.70, −0.46) | <0.01 |
| CRT > 400 | −162.15 (−207.50, −116.79) |  | −54.46 (−109.35, 0.43) | 0.05 | −160.71 (−142.62, −70.80) | <0.01 |
| CRT ≤ 400 | [Reference] |  | [Reference] |  | [Reference] |  |
| MRT (μm) | −0.59 (−0.72, −0.45) | <0.01 | −0.29 (−0.45, −0.13) | <0.01 | −0.43 (−0.53, −0.32) | <0.01 |
| MRT > 400 | −113.24 (−106.57, −56.91) | <0.01 | −84.69 (−143.50, −25.89) | 0.01 | −4.30 (−49.74, 41.14) | 0.85 |
| MRT ≤ 400 | [Reference] |  | [Reference] |  | [Reference] |  |
| SFCT (μm) | −0.26 (−0.71, 0.19) | 0.26 | 0.23 (−0.20, 0.65) | 0.30 | 0.01 (−0.30, 0.32) | 0.96 |
| Presence of IRF^b^ | −59.56 (−112.90, −6.22) | 0.03 | −26.88 (−85.95, 32.19) | 0.37 | −43.53 (−82.93, −4.12) | 0.03 |
| Presence of SRF^b^ | −73.57 (156.30, 9.16) | 0.08 | −49.48 (−201.70, 102.75) | 0.52 | −63.39 (−135.95, 9.17) | 0.09 |
| SRF within 1-mm central subfield^b^ | −14.75 (−67.11, 37.61) | 0.58 | −22.25 (−78.83, 34.33) | 0.44 | −17.93 (−56.06, 20.20) | 0.36 |
| PED within 1-mm central subfield^b^ | −10.95 (−63.48, 41.59) | 0.68 | 28.58 (−27.91, 85.07) | 0.32 | 8.68 (−29.53, 46.90) | 0.66 |
| PED maximum height (μm) | 0.07 (−0.01, 0.23) | 0.41 | −0.01 (−0.15, 0.13) | 0.88 | 0.02 (−0.08, 0.13) | 0.68 |
| PED volume (mm^3^) | 21.06 (−12.56, 54.68) | 0.22 | −3.28 (−32.43, 25.88) | 0.82 | 6.69 (−15.00, 28.38) | 0.54 |
| PED types, no. (%)  I (Fibrovascular PED)  II (Hemorrhagic PED)  III (Serous vascularized PED) | [Reference]  −66.74 (−135.44, 1.97)  None | 0.06 | [Reference]  −46.99 (−108.71, 14.72)  None | 0.13 | [Reference]  −53.01 (−98.22, −7.96)  None | 0.02 |
| PED shapes, no. (%)  Dome-shaped  Multilobular  Sharp peaked  Shallow irregular  Suspect RPE tear | [Reference]  −9.26 (−80.73, 62.20)  3.25 (−67.52, 74.02)  −5.10 (−97.50, 87.30)  147.00 (−63.26, 357.26) | 0.80  0.93  0.91  0.17 | [Reference]  11.02 (−61.46, 83.50)  −25.21 (−105.08, 54.67)  −91.43 (−227.03, 44.17)  109.15 (40.18, 178.12) | 0.76  0.53  0.18  <0.01 | [Reference]  48.43 (−1.91, 98.76)  8.39 (−42.69, 59.47)  −17.82 (−79.16, 43.52)  −26.57 (−141.97, 88.82) | 0.06  0.75  0.57  0.65 |
| Polypoidal lesions number (no.) | 8.03 (−2.45, 18.52) | 0.13 | −5.81 (−17.55, 5.94) | 0.33 | 1.57 (−6.25, 9.38) | 0.69 |
| Polypoidal lesions area (mm^2^) | 150.44 (5.42, 295.46) | 0.04 | −69.26 (−196.13, 57.60) | 0.28 | 23.48 (−71.52, 118.48) | 0.63 |
| Polypoidal lesions within 1-mm central subfield^b^ | 5.05 (−47.06, 57.15) | 0.19 | 3.33 (−50.87, 57.52) | 0.90 | 4.23 (−33.00, 41.45) | 0.82 |
| Presence of BVN^b^ | 76.58 (−128.51, 281.67) | 0.46 | 8.77 (−272.69, 290.22) | 0.95 | 54.70 (−108.89, 218.28) | 0.51 |
| BVN area (mm^2^) | 5.51 (−2.64, 13.65) | 0.18 | 2.84 (−2.70, 8.38) | 0.32 | 3.75 (−0.80, 8.30) | 0.11 |
| Presence of retinal hemorrhage^b^ | −13.50 (−72.84, 45.37) | 0.65 | −39.04 (−95.32, 17.25) | 0.17 | −25.89 (−66.04, 14.26) | 0.21 |
| Retinal hemorrhage area (mm^2^) | −3.41 (−11.96, 5.14) | 0.43 | −1.51 (−7.52, 4.51) | 0.62 | −1.98 (−6.79, 2.83) | 0.42 |
| Retinal hemorrhage > 1 DD^b^ | 13.12 (−59.66, 85.89) | 0.72 | −52.36 (−116.17, 11.46) | 0.11 | −23.35 (−70.70, 24.00) | 0.33 |
| Retinal hemorrhage within 1-mm central subfield^b^ | 12.93 (−70.80, 96.65) | 0.76 | 24.55 (−79.22, 128.32) | 0.64 | 16.77 (−48.06, 81.60) | 0.61 |
| Presence of CVH^b^ | 4.57 (−58.25, 67.39) | 0.89 | 6.98 (−52.19, 66.14) | 0.82 | 4.60 (−37.72, 46.92) | 0.83 |
| CNV within 1-mm central subfield^b^ | −127.37 (−223.93, −30.60) | 0.01 | 44.62 (−92.57, 181.80) | 0.52 | −65.75 (−145.52, 14.01) | 0.11 |
| GLD (μm) | 0.01 (−0.01, 0.03) | 0.27 | 0.01 (−0.01, 0.03) | 0.53 | 0.01 (−0.01, 0.02) | 0.23 |

3+Q12W = 3 monthly injections followed by injections every 12 weeks; 3+TAE = 3 monthly injections followed by treat and extend regimen; BCVA = best-corrected visual acuity; BVN = branching vascular network; CI = confidence interval; CNV = choroidal neovascularization; CRT = central retinal thickness; CVH = choroidal vascular hyperpermeability; DD = disk diameter; ETDRS = Early Treatment Diabetic Retinopathy Study; GLD = greatest linear dimension; IRF = intraretinal fluid; MRT = maximum retinal thickness; PED = pigment epithelial detachment; RPE = retinal pigment epithelium; SFCT = subfoveal choroidal thickness; SRF = subretinal fluid.

^b^ In unvariate regression analysis, we used "0" to indicate absence and "1" to indicate presence.

**Supplementary Table 4.** Univariate Analysis of the change in MRT at Week 48 for each Candidate Predictive Factor by Treatment Group

| Change in MRT from baseline at week 48 | 3+TAE | | 3+Q12W | | All | |
| --- | --- | --- | --- | --- | --- | --- |
|  | Unstandardized coefficients B  (95% CI) | *P* value | Unstandardized coefficients B  (95% CI) | *P* value | Unstandardized coefficients B  (95% CI) | *P* value |
| Gender (Women = 0) | −13.30 (−79.09, 52.48) | 0.69 | 30.28 (−41.69, 102.24) | 0.41 | 8.73 (−39.58, 57.04) | 0.72 |
| Age | −0.94 (−5.44, 3.56) | 0.68 | 0.65 (−4.16, 5.45) | 0.79 | −0.15 (−3.41, 3.12) | 0.93 |
| Treatment regimen  (3+TAE = 0 and 3+Q12W = 1) |  |  |  |  | 20.86 (−26.02, 67.73) | 0.38 |
| BCVA (ETDRS letters) | 1.89 (−0.31, 4.09) | 0.09 | 3.29 (0.87, 5.71) | 0.01 | 2.58 (0.96, 4.20) | <0.01 |
| BCVA < 34 | −134.18 (−290.38, 22.02) | 0.09 | −131.45 (−335.21, 72.30) | 0.20 | −140.19 (−262.50, 17.89) | 0.03 |
| 34 ≤ BCVA ≤ 73 | −38.73 (−123.73, 46.27) | 0.37 | −66.20 (−149.44, 17.04) | 0.12 | −54.51 (−113.27, 4.26) | 0.07 |
| BCVA > 73 | [Reference] |  | [Reference] |  | [Reference] |  |
| CRT (μm) | −0.71 (−0.90, −0.52) | <0.01 | −0.33 (−0.55, −0.11) | <0.01 | −0.57 (−0.41, −0.74) | <0.01 |
| CRT > 400 | −157.64 (−219.76, −95.52) | <0.01 | −42.43 (−113.38, 28.52) | 0.24 | −97.75 (−145.08, −50.43) | <0.01 |
| CRT ≤ 400 | [Reference] |  | [Reference] |  | [Reference] |  |
| MRT (μm) | −0.73 (−0.90, −0.55) | <0.01 | −0.40 (−0.60, −0.19) | <0.01 | −0.55 (−0.68, −0.41) | <0.01 |
| MRT > 400 | −137.25 (−209.08, −65.41) | <0.01 | −112.68 (−187.73, −37.64) | <0.01 | −18.30 (−77.93, 41.32) | 0.55 |
| MRT ≤ 400 | [Reference] |  | [Reference] |  | [Reference] |  |
| SFCT (μm) | −0.16 (−0.74, 0.41) | 0.58 | 0.23 (−0.32, 0.77) | 0.41 | 0.05 (−0.34, 0.44) | 0.79 |
| Presence of IRF^b^ | −51.57 (−119.93, 16.78) | 0.14 | −45.32 (−120.72, 30.09) | 0.24 | −47.98 (−98.37, 2.41) | 0.06 |
| Presence of SRF^b^ | −12.60 (−118.80, 93.60) | 0.82 | −10.04 (−205.05, 184.97) | 0.92 | −5.94 (−99.00, 87.13) | 0.90 |
| SRF within 1-mm central subfield^b^ | 28.19 (−37.94, 94.31) | 0.40 | 24.73 (−47.68, 97.14) | 0.50 | 27.33 (−21.19, 75.84) | 0.27 |
| PED within 1-mm central subfield^b^ | −20.22 (−86.81, 46.37) | 0.55 | 37.08 (−35.15, 109.32) | 0.31 | 8.57 (−40.16, 57.29) | 0.73 |
| PED maximum height (μm) | 0.03 (−0.18, 0.24) | 0.77 | −0.07 (−0.25, 0.11) | 0.44 | −0.03 (−0.16, 0.11) | 0.70 |
| PED volume (mm^3^) | 9.53 (−33.31, 52.38) | 0.66 | −15.66 (−52.54, 21.23) | 0.40 | −4.98 (−32.49, 22.54) | 0.72 |
| PED types, no. (%)  I (Fibrovascular PED)  II (Hemorrhagic PED)  III (Serous vascularized PED) | [Reference]  −95.42 (−182.21, −8.64)  None | 0.03 | [Reference]  −106.64 (−183.97, −29.32)  None | 0.01 | [Reference]  −97.6 (−154.44, −40.72)  None | <0.01 |
| PED shapes, no. (%)  Dome-shaped  Multilobular  Sharp peaked  Shallow irregular  Suspect RPE tear | [Reference]  −1.09 (−91.81, 89.63)  17.97 (−71.87, 107.82)  8.92 (−108.37, 126.21)  180.52 (−86.40, 447.44) | 0.98  0.69  0.88  0.18 | [Reference]  170.58 (83.93, 257.22)  54.45 (−36.61,145.51)  7.80 (−92.54, 108.15)  −131.12 (−301.48, 39.24) | <0.01  0.24  0.88  0.13 | [Reference]  82.78 (18.87, 146.68)  36.48 (−28.36, 101.32)  6.55 (−71.31, 84.41)  −47.75 (−194.23, 98.73) | 0.01  0.27  0.87  0.52 |
| Polypoidal lesions number (no.) | 11.44 (−1.83, 24.70) | 0.09 | −5.88 (−21.43, 9.66) | 0.46 | 3.31 (−6.82, 13.44) | 0.52 |
| Polypoidal lesions area (mm^2^) | 251.19 (69.56, 432.82) | 0.01 | −118.92 (−285.67, 47.84) | 0.16 | 39.70 (−83.38, 162.78) | 0.53 |
| Polypoidal lesions within 1-mm central subfield^b^ | −2.71 (−68.97, 63.55) | 0.94 | 7.41 (−64.06, 78.88) | 0.84 | 2.31 (−45.97, 50.59) | 0.93 |
| Presence of BVN^b^ | 88.80 (−172.45, 350.06) | 0.50 | 12.73 (−368.12, 393.58) | 0.95 | 66.28 (−148.55, 281.11) | 0.54 |
| BVN area (mm^2^) | 4.62 (−5.79, 15.03) | 0.38 | 2.28 (−5.24, 9.79) | 0.55 | 3.20 (−2.80, 9.19) | 0.30 |
| Presence of retinal hemorrhage^b^ | −31.50 (−106.31, 43.32) | 0.41 | −24.55 (−96.96, 47.86) | 0.50 | −24.77 (−76.04, 26.49) | 0.34 |
| Retinal hemorrhage area (mm^2^) | −3.50 (−14.36, 7.35) | 0.52 | −0.92 (−8.63, 6.79) | 0.81 | −1.47 (−7.62, 4.68) | 0.64 |
| Retinal hemorrhage > 1 DD^b^ | 11.77 (−80.55, 104.08) | 0.80 | −50.70 (−132.69, 31.29) | 0.22 | −21.46 (−81.89, 38.96) | 0.49 |
| Retinal hemorrhage within 1-mm central subfield^b^ | 58.31 (−47.41, 164.03) | 0.28 | 70.47 (−61.76, 202.70) | 0.29 | 61.26 (−21.09, 143.61) | 0.14 |
| Presence of CVH^b^ | 12.06 (−67.35, 91.47) | 0.76 | −0.39 (−76.18, 75.41) | 0.99 | 2.90 (−51.00, 56.80) | 0.92 |
| CNV within 1-mm central subfield^b^ | −144.41 (−267.77, −21.05) | 0.02 | 96.29 (−78.70, 271.28) | 0.28 | −59.47 (−161.51, 42.58) | 0.25 |
| GLD (μm) | 0.01 (−0.02, 0.04) | 0.54 | 0.01 (−0.02, 0.03) | 0.70 | 0.01 (−0.01, 0.03) | 0.48 |

3+Q12W = 3 monthly injections followed by injections every 12 weeks; 3+TAE = 3 monthly injections followed by treat and extend regimen; BCVA = best-corrected visual acuity; BVN = branching vascular network; CI = confidence interval; CNV = choroidal neovascularization; CRT = central retinal thickness; CVH = choroidal vascular hyperpermeability; DD = disk diameter; ETDRS = Early Treatment Diabetic Retinopathy Study; GLD = greatest linear dimension; IRF = intraretinal fluid; MRT = maximum retinal thickness; PED = pigment epithelial detachment; RPE = retinal pigment epithelium; SFCT = subfoveal choroidal thickness; SRF = subretinal fluid.

^b^ In unvariate regression analysis, we used "0" to indicate absence and "1" to indicate presence.
